# Roadway rock burst prediction based on catastrophe theory

**DOI:** 10.1038/s41598-024-58072-0

**Published:** 2024-03-27

**Authors:** Wang Pan, Gu Shuan-Cheng, Sun Wei

**Affiliations:** School of Architecture and Civil Engineering, Xi’an University of Science and Technology, Xi’an, 710054 China

**Keywords:** Rock burst, Key load-bearing circle of surrounding rock, Cusp catastrophe, Critical load, Critical depth, Energy science and technology, Engineering, Mathematics and computing

## Abstract

In order to quantitatively calculate the critical depth and critical load of mines affected by rock burst, and to achieve effective prevention and control of rock burst in coal mines, this paper proposes a mechanical model for predicting the occurrence of rock burst in coal mine roadways based on catastrophe theory. Additionally, a theoretical calculation formula for initiating rock burst is derived. The first step was to establish a mechanical analysis model, which directly correlated with the in-situ stress, physical and mechanical characteristics of the coal-rock mass, and engineering structural parameters. Following this, a mechanical instability criterion was derived for the key load-bearing circle within the surrounding rock of the roadway. In the final step, the critical depth and load for rock burst initiation were verified for 25 distinct coal mines in China that were prone to rock burst hazards. The research results demonstrate that the discrepancy between the theoretically calculated critical depth and the actual measured statistical values was less than 35%. In addition, the difference between the theoretically determined critical depth and the value calculated by Pan Yishan was less than 32%. Notably, the ratio of the theoretically calculated critical load to the uniaxial compressive strength of the coal-rock mass ranged from 0.38 to 1.93. This aligns with empirical data on rock burst occurrences, as set out in the engineering classification standards for rock masses. These research outcomes substantiated the practical utility of the proposed theory, thereby laying a robust theoretical groundwork for the quantitative control of rock burst.

## Introduction

In the coal mining process, five types of natural hazards are commonly encountered: water, fire, gas outbursts, dust, and roof falls, which are collectively referred to as the "five major disasters"^1–7^. The phenomenon of rock burst, as a unique form of strata pressure behavior, has become a predominant hazard in coal mining, especially in deep mining operations, posing a grave threat to the safety of coal production. In recent years, with the increase in mining depth and intensity, rock bursts in coal mines have become increasingly severe and complex, causing heavy casualties and economic losses^8^. On October 20, 2018, a roadway burst at the Longyan Coal Mine in Yancheng City caused the closure of a 100-m roadway and killed 21 miners. On June 9, 2019, another incident killed nine people at the Longjiapu coal mine in Jilin Province, China. On October 11, 2021, a major crash struck the Hujiahe coal mine in Shaanxi province, China, killing four people.

The frequent occurrences of major rock burst disasters have garnered high-level attention from national leaders. They have repeatedly issued directives emphasizing the need for thorough research into the root causes of rock burst and for the implementation of effective solutions. In 2020, the State Council's Work Safety Committee issued a notice titled "On Further Implementing General Secretary Xi Jinping’s Important Instructions and Resolutely Preventing and Controlling Coal Mine Rock burst Accidents"^9^. This notice highlighted the importance of reinforcing the objective of achieving "zero rock burst" (no casualties, no tunnel damage, and no equipment damage) in coal and rock mining operations, strictly managing and controlling occurrences and incidents of rock burst, and resolutely curbing the occurrence of accidents. Consequently, predicting rock burst and their prevention and control has become a crucial aspect in safeguarding the safety of coal mine production.

In the event of a rock burst, the coal-rock mass experiences a sudden, sharp, and intense release of strain energy, often leading to the instantaneous destruction of the coal-rock layer structure within the working face or roadway^2^. Mines with coal seams susceptible to rock burst are known as rock burst mines. For example, if a mine starts to experience rock burst after reaching a certain depth, this threshold is considered the critical depth specific to that rock burst mine^10^. The critical depth varies based on geological conditions; however, the general trend suggests that the risk of rock burst increases with mining depth and intensity^11^. Consequently, quantitatively determining the critical depth at which a rock burst occurs is particularly important for their effective prevention and control. In addressing this, Hu et al.^12^ explored issues faced during deep mining in China's coal mines—such as high geothermal conditions, rock burst, gas emissions, Ordovician limestone water inrush, and mining-induced effects—and proposed the concept and classification principles for deep mines. Li et al.^13^, by constructing an ultra-low friction type rock burst block model, studied the specific impacts of rock burst load intensity and mining depth, identifying critical depth zones for rock burst at 400–600, 800–1000, and 1200 m. Qi et al.^2^ investigated the occurrence mechanism and control technology of rock burst, suggesting that the critical depths for Chinese mines range from 200 to 540 m, with an average depth of 380 m. Additionally, Qin et al.^14^ used UDEC discrete element simulation software for numerical simulations to evaluate the effects of roadway burial depth and disturbance stress intensity on the stability of surrounding rock, establishing critical depths and critical load intensities for the onset of rock burst. However, to date, there is no consensus on the critical depth for rock burst mines, as research findings present a variety of depth ranges. Furthermore, rock burst in coal mines involves the throwing or overall displacement of a coal body within a certain range of the roadway (the thrown part of the coal body is turned into a shock body), and rock burst disasters are inevitably closely related to the instability in near-field surrounding rock^15^. Despite this, current modeling studies have yet to define the range of the shock bodies^3,4^.

Therefore, this paper analyzes the rock burst characteristics in coal mine roadways and views the surrounding rock model of the roadway as a three-area structure: "elastic area," "plastic area," and "broken area." It defines the surrounding rock in the broken area as the shock body and regards the surrounding rocks within the plastic area as the key bearing ring. For the first time, this study establishes a key bearing ring instability critical point mutation model for mine roadways subjected to shock pressure, derives mechanical criteria for the instability of the key bearing ring, and determines the critical load and depth for the initiation of rock burst. Moreover, the model combines the stress increment mutation criterion of surrounding rock with catastrophe theory to establish a prediction model for rock burst and derive a stability discrimination formula for the roadway bearing ring. Combined with numerical simulations and practical engineering analysis, the research results offer a new approach to the quantitative prediction of rock burst.

### Cusp catastrophe model for roadway rock burst prediction

#### Mechanistic simplified model

**Figure 1:**
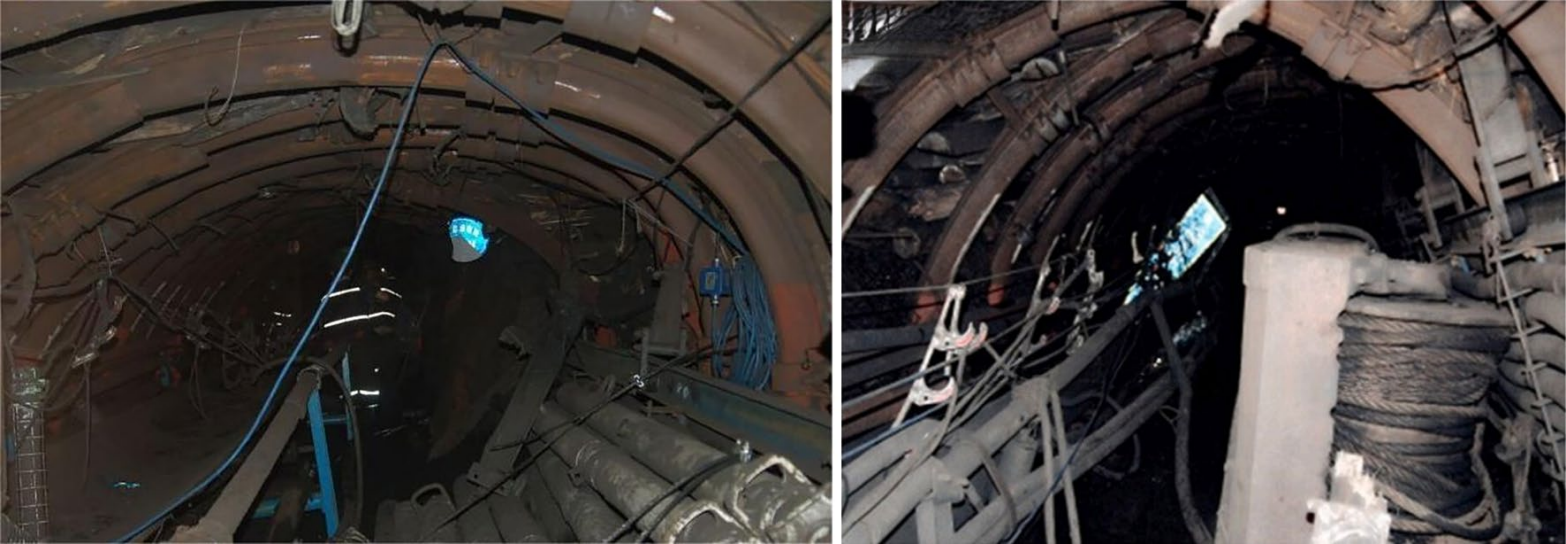
In-situ rock burst in a roadway.

Once the roadway is excavated, the stress in the primary rock is alleviated, leading to a redistribution of stress within the surrounding rock sections. Consequently, the radial stress around the roadway reduces to zero, while the tangential stress becomes more concentrated. When the concentrated stress exceeds the strength of the surrounding rock, the rock around the roadway undergoes destruction. This results in a gradual increase in depth until a new three-way stress balance is reached at a certain depth. The primary load-bearing zone of the surrounding rock, which encompasses the lithosphere bearing significant tangential stress within a specific range of the roadway, is vital for maintaining the stability of the roadway^16,17^. The actual observation demonstrates that when subjected to ground pressure, the key load-bearing circle rock block undergoes strain-induced rotation and produces slight sliding between adjacent blocks. This sliding and rotation are stabilized to a certain extent by the interaction of nearby rocks. In this way, the area of some blocks of the key load-bearing circleis tens of times larger than the displacement caused by the block strain. Therefore, the key load-bearing circleis prone to become a flat ellipse under a large vertical force and a vertical ellipse under a large lateral force as shown in Fig. 1. However, under a large hydrostatic pressure, the instability in the rock block in the key load-bearing circle can lead to instability in the entire key load-bearing circle. Therefore, the mechanical behaviour of the key load-bearing circle before the instability is similar to that of an elastomer with low stiffness. Therefore, to study its stability, the thicker key load-bearing circle was reduced to a thinner elastic ring after considering its unit width.

The model assumptions are as follows:

1. The object of analysis for coal mine rock burst is abstracted as the most typical circular tunnel.
2. Tectonic stress is not considered; thus, the boundary load is simplified to uniform hydrostatic pressure, namely, the primary rock stress $P_{0}$.
3. The model is divided into a three-zone structure:"elastic area, plastic area, and broken area". The surrounding rock of the broken area is defined as the shock body, while the surrounding rock within the plastic area serves as the key bearing ring.

**Figure 2:**
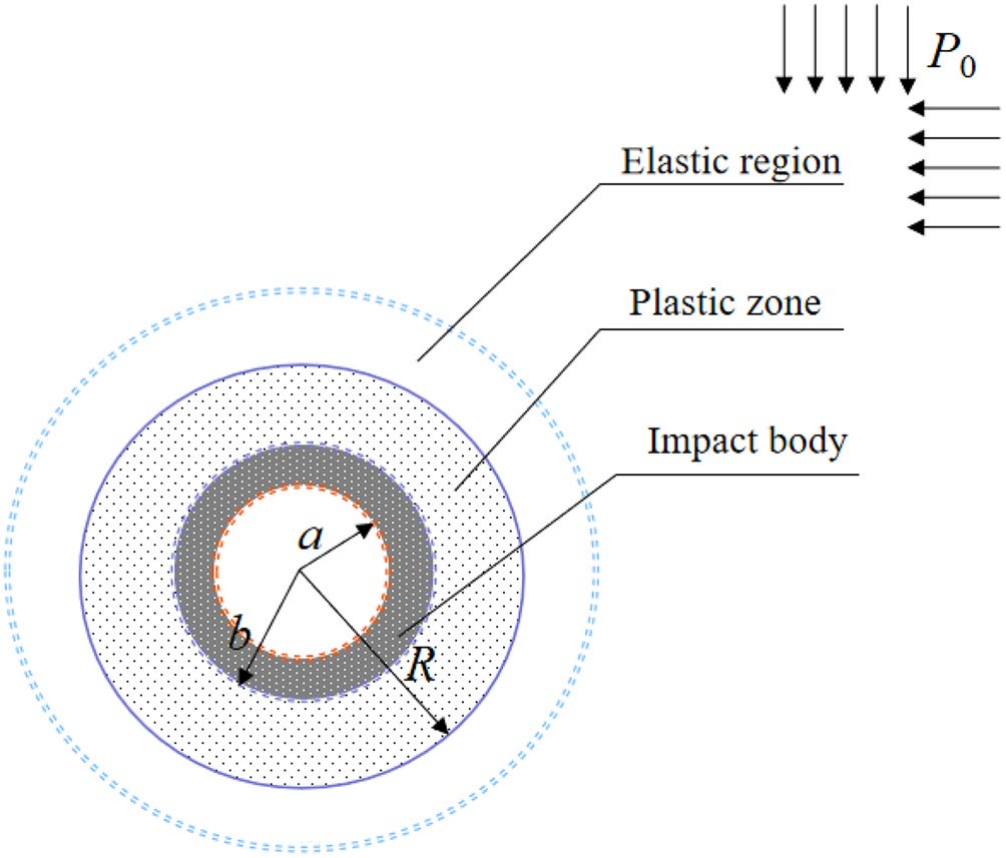
Schematic diagram of roadway surrounding rock partition.

**Figure 3:**
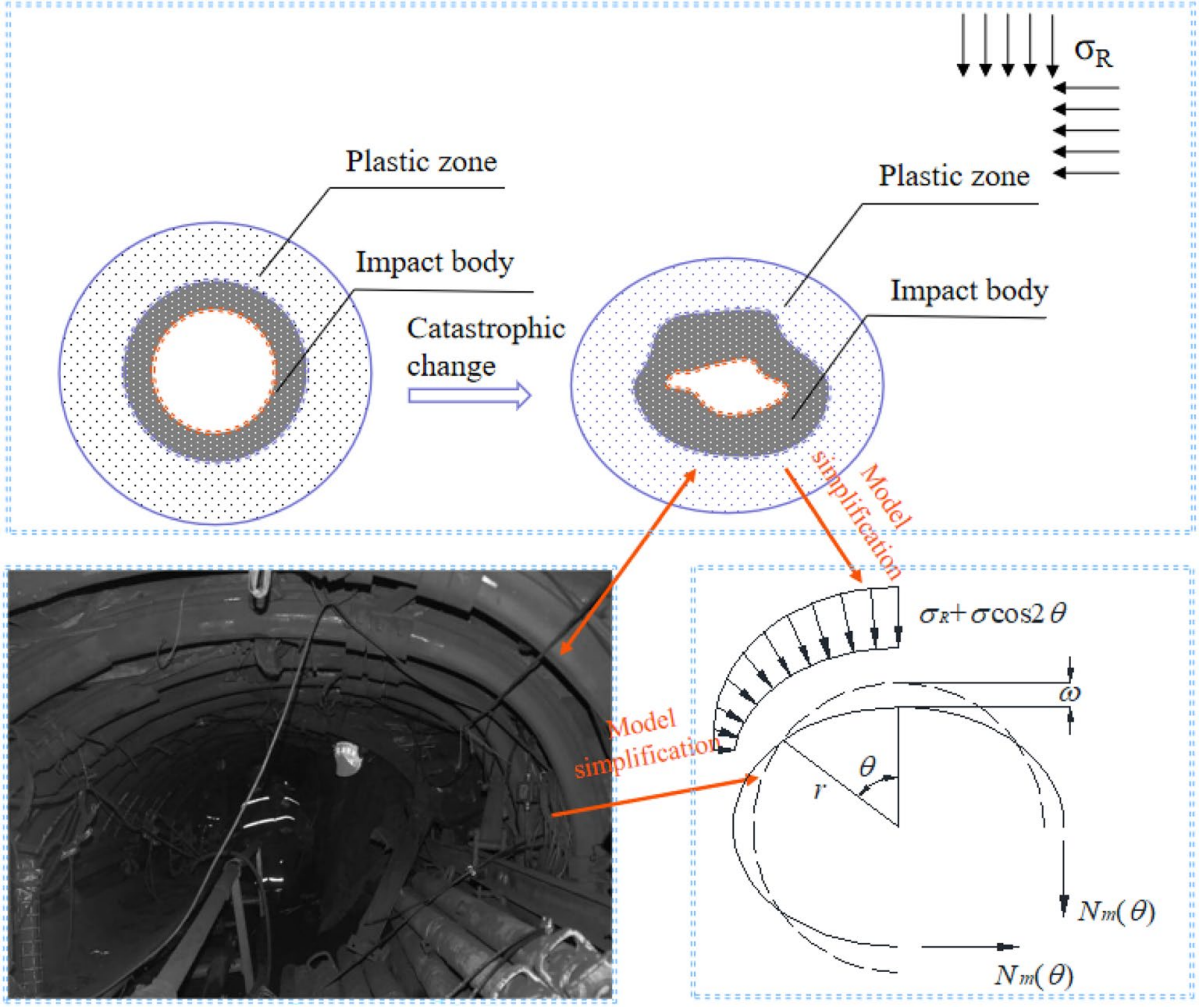
Impact failure diagram of a surrounding rock in roadway.

The schematic diagram of the model zoning is shown in Fig. 2. The occurrence of rock burst events can be attributed to the instability in the structure of the key load-bearing circle, which is caused by the elastic area load. This instability leads to the sudden displacement of the surrounding rock in the broken area. In other words, dynamic failure of the impact body occurs, as shown in Fig. 3.

#### Cusp catastrophe model

**Figure 4:**
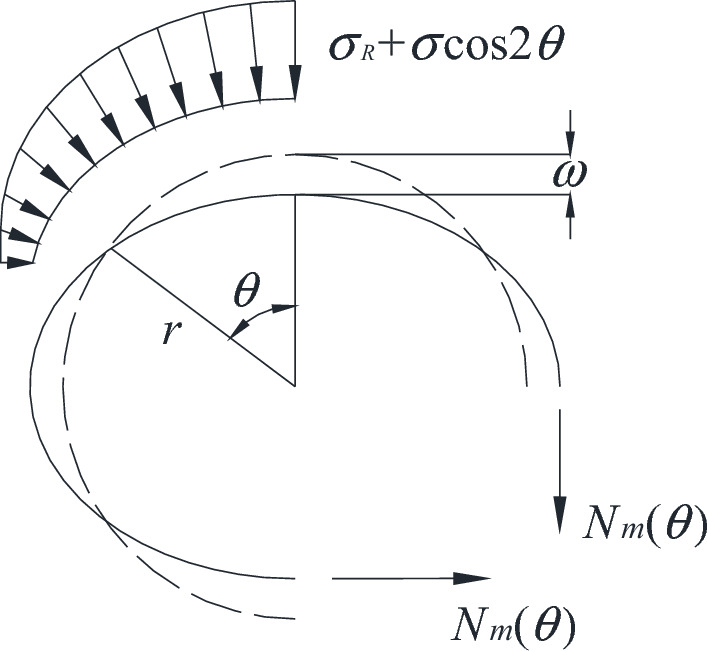
Middle layer displacement of the ring under load.

As shown in Fig. 4, the solid and dashed lines indicate the position of the elastomer after and before deformation, respectively. The force and displacement of the ring are as follows.

Here, $N_{m}$ (N)is the circumferential force.

1 $$ \sigma_{\theta } = \sigma_{R} + \sigma \cos 2\theta , $$

2 $$ \sigma_{R} = C\cot \phi (\frac{R}{a})^{{\frac{2\sin \phi }{{1 - \sin \phi }}}} - C\cot \phi , $$

where $\sigma_{R}$ (MPa) is the radial stress at the elastic and plastic interface of the surrounding rocks, $\sigma \cos 2\theta$ (MPa) is the distributed force that varies with $\theta$ (°) (The distribution force exhibits symmetry along both the horizontal and vertical axes, representing the additional force resulting from external factors acting on the elliptical circumference. As a result, the stress primarily is $\sigma_{R}$), $C$ (MPa) is the cohesion, and $\phi$ (°) is the internal friction angle .

3 $$ w = \omega \cos 2\theta , $$

where $w$ (mm) is the circular ring axis displacement.

The key load-bearing circle, which consists of the plastic zone in the surrounding rock, serves as the basis for establishing the catastrophe model of this system.The total potential energy $W$ (J) is

4 $$ W = W_{b} + W_{m} + W_{p} , $$

where $W_{b}$ (J)is the bending strain energy of the load-bearing circle, $W_{m}$ (J) is the circumferential force strain energy of the load-bearing circle, $W_{p}$ (J)is the loading potential energy of the load-bearing circle.

The bending strain energy of the ring adopts the calculation formula of the beam as follows.

5 $$ W_{b} = \int_{0}^{2\pi } {\frac{{M^{2} }}{2EI}} rd\theta , $$

where $I = t^{3} /12$ (mm^4^) is the section moment of the unit width circle, $E$ (MPa) is the elastic modulus, $t$ (mm) is the thickness of the key load-bearing circle, $M$ (N·mm) is the bending moment, and $r$ (mm) is the middle layer radius of the the key load-bearing circle.

6 $$ r = \frac{R + b}{2}, $$

7 $$ R = a\left[ {\frac{{(p_{0} + C\cot \phi )(1 - \sin \phi )}}{C\cot \phi }} \right]^{{\frac{1 - \sin \phi }{{2\sin \phi }}}} , $$

where $p_{0}$ (MPa) is the primary rock stress, and $a$ (mm) is the radius of the roadway.

The scope of the crushing area is defined as the surrounding rock near the roadway whose stress is less than that of the stress of the primary rock. The radius of the crushing area is

8 $$ b = a\left[ {\frac{{(p_{0} + C\cot \phi )(1 - \sin \phi )}}{C\cot \phi (1 + \sin \phi )}} \right]^{{\frac{1 - \sin \phi }{{2\sin \phi }}}} . $$

Based on Eq. (8), the volume of the shock body can be calculated, thereby quantitatively describing the severity of the rock burst.

The inner ring bending moment $M$ can be expressed as

9 $$ M = - \left( {\frac{1}{{r^{\prime}}} - \frac{1}{r}} \right)EI, $$

where $1/r^{\prime}$ (mm^-1^) is the curvature of the middle layer of the ring after the deformation.

The geometric relation between $r$, $w$, and the curvature of the middle layer of the ring after deformation is

10 $$ \frac{1}{{r^{\prime}}} = (\frac{1}{{r^{\prime}}} + \frac{1}{{r^{2} }}\frac{{d^{2} w}}{{d\theta^{2} }})/(1 - \frac{w}{r}). $$

By substituting Eqs. (3), (9), and (10) into Eq. (5), the following is obtained:

11 $$ W_{b} = \frac{81EI\pi }{{8r}}\left( {\frac{\omega }{r}} \right)^{4} . $$

The strain energy generated by the circumferential force $N_{m}$ is

12 $$ W_{m} = \int_{0}^{2\pi } \frac{1}{2} N_{m} \left( {\frac{{d^{2} w}}{{r^{2} d\theta^{2} }} + \frac{w}{{r^{2} }}} \right)wrd\theta . $$

The circumferential force $N_{m}$ can be obtained according to the equilibrium method

13 $$ N_{m} = r\left( {\sigma_{R} + \frac{\sigma }{3}\cos 2\theta } \right). $$

The load potential energy $W_{p} = - T$, $T$(J) is the work done by the external forces, so $W_{p}$ is

14 $$ W_{p} = - T = - \int_{0}^{2\pi } {\frac{1}{2}(\sigma_{R} + \sigma \cos 2\theta )} w \cdot rd\theta = - \frac{{r^{2} \pi \sigma }}{2}\left( \frac{w}{r} \right). $$

By substituting Eqs. (11), (12), (13), and (14) into Eq. (4), the following is obtained:

15 $$ W = \frac{81}{8}\frac{EI\pi }{r}\left( {\frac{\omega }{r}} \right)^{4} + \frac{3}{2}r^{2} \pi \left( {\frac{3EI}{{r^{3} }} - \sigma_{R} } \right)\left( {\frac{\omega }{r}} \right)^{2} - \frac{{r^{2} \pi \sigma }}{2}\left( {\frac{\omega }{r}} \right). $$

Equation (15) can be changed as follows.

16 $$ W = \frac{{x^{4} }}{4} + \frac{d}{2}x^{2} + ex, $$

where $x = \left( {\frac{81EI\pi }{{2r}}} \right)^{\frac{1}{4}} \left( {\frac{\omega }{r}} \right)$; $d$ is the control variable, $d = 3r^{2} \pi \left( {\frac{3EI}{{r^{3} }} - \sigma_{R} } \right) \cdot \left( {\frac{2r}{{81EI\pi }}} \right)^{\frac{1}{2}}$; and $e$ is the control variable, $e = - \frac{{r^{2} \pi }}{2}\sigma \left( {\frac{2r}{{81EI\pi }}} \right)^{\frac{1}{4}} .$

Equation (16) is the standard form of the cusp catastrophe model. According to $\frac{d}{dx}W(x) = 0$ and $\frac{{d^{2} }}{{dx^{2} }}W(x) = 0$, the bifurcation point set in Eq. (17) can be obtained. At the same time, the balance surface and bifurcation curve diagram are obtained. The bifurcation curve is the projection of all catastrophe points on the balance surface on the control plane, as shown in Fig. 4.

17 $$ \Delta = 4d^{3} + 27e^{2} $$

**Figure 5:**
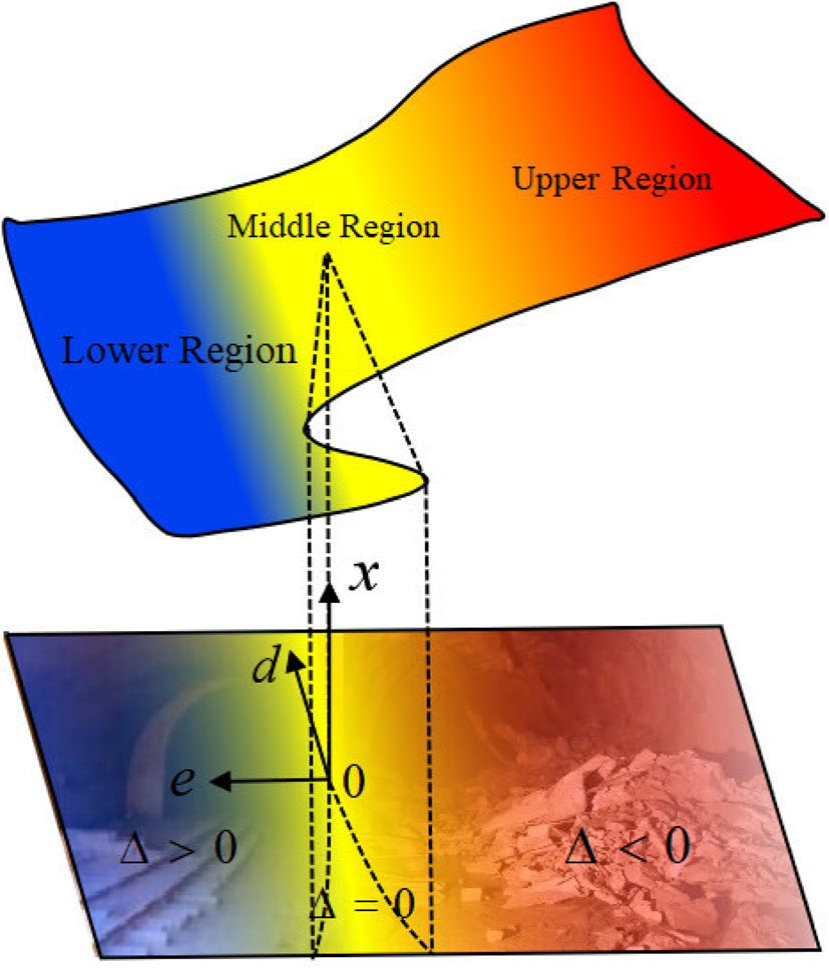
Equilibrium surface and branching curve of the circular ring catastrophe model under $\sigma_{R} + \sigma \cos 2\theta$.

Figure 5 illustrates the cusp catastrophe model. The surface depicted in this figure is the system's equilibrium surface, which is a smooth but folded surface within three-dimensional space. This surface can be divided into upper, middle, and lower regions, each representing a different equilibrium position. Both the upper and lower regions are stable, while the middle region is unstable. Within the middle region, there is a fold area that forms a set of points on a plane, known as the bifurcation set. When the parameter $d$ is positive ($\Delta > 0$), if a phase point moves from the upper to the lower region of the equilibrium surface, stable changes in $d$ and $e$ almost always cause $x$ to change steadily, and at this time the bearing ring system remains stable, with no abrupt transformation occurring. However, when $d$ is negative ($\Delta < 0$), if a phase point is precisely in the fold area of the surface, it will inevitably leap from the upper to the lower region, consequently causing an abrupt change in the parameter $x$, at which point the bearing ring system becomes unstable, resulting in a rock burst. The cusp catastrophe model effectively characterizes the occurrence of a rock burst: as the mining system enters the bifurcation set region, any disturbance can suddenly change the system's state, leading to severe destruction. The principles of catastrophe theory, with its focus on immediacy and suddenness, align well with the nature of a rock burst, making it a suitable tool for studying the mechanisms behind the occurrence of rock bursts.

#### Mechanical instability criterion

Figure 5 reveals that the condition $\Delta = 0$ is crucial for determining the stability of the key load-bearing circle system in the surrounding rock. It serves as both a necessary and sufficient condition for instability. However, this condition can only be met when the system crosses the bifurcation set with $d \le 0$, leading to a transition of the load-bearing ring system from a state of equilibrium to one of nonequilibrium. A bifurcation set equation equal to zero is difficult to attain, and also the parameter calculation is complex. For practical convenience and construction safety, $d \le 0$ is the mechanical criterion used to determine if the project's system exhibits catastrophe and instability. Substituting Eq. (17) into $d \le 0$ yields the mechanical criterion for the instability of the key load-bearing circle system in the surrounding rock.

18 $$ d = 3r^{2} \pi \left( {\frac{3EI}{{r^{3} }} - \sigma_{R} } \right) \cdot \left( {\frac{2r}{{81EI\pi }}} \right)^{\frac{1}{2}} \le 0, $$

19 $$ \frac{3EI}{{r^{3} }} - \sigma_{R} \le 0. $$

Assuming that the circle section is unchanged during bending, considering the plane strain state, so $E/(1 - \mu^{2} )$ should replace $E$ in Eq. (19):

20 $$ \frac{2E}{{1 - \mu {}^{2}}}(\frac{t}{2r})^{3} \le \sigma_{R} . $$

Substituting Eqs. (2), (7), and (8) into Eq. (20), the critical load for rock burst initiation can be obtained as

21 $$ P_{{{\text{cr}}}} = \sigma_{{\text{c}}} \eta , $$

where $$\eta = \left\{ {\frac{E}{{(1 - \mu^{2} )C{\text{cos}}\phi }}\left[ {\frac{{(1 + \sin \phi )^{{\frac{1 - \sin \phi }{{2\sin \phi }}}} - 1}}{{(1 + \sin \phi )^{{\frac{1 - \sin \phi }{{2\sin \phi }}}} + 1}}} \right]^{3} + \frac{\sin \phi }{2}} \right\}$$.

In a typical scenario, mining at greater depths imposes increased stress on the coal body. This exacerbates deformation and prompts an accumulation of elastic energy within the coal body, thereby augmenting the propensity for rock burst events. Absent consideration for structural stress, and given that the bulk density of the seam's overlying strata is represented by $\gamma$ (kg/mm^3^), with the roadway depth denoted as $H$ (mm), the far-field ground stress generated by the overburden pressure is expressed as $P_{0} = \gamma H$. Upon reaching critical state for initiating a rock burst, the critical mining depth corresponding to rock burst occurrence becomes

22 $$ H_{{{\text{cr}}}} = \frac{{P_{{{\text{cr}}}} }}{\gamma }. $$

The stability coefficient of the key load-bearing circle system is defined as

23 $$ K = \frac{{\sigma_{{\text{c}}} \eta }}{{P_{{0}} }}. $$

Based on Eq. (23), when $K \le 1$, the key load-bearing circle system of the surrounding rock enters an unstable equilibrium. This instability subsequently leads to the release of accumulated elastic strain energy within the elastic area. As a result, there is a sudden discharge of the surrounding rock in the broken area. This phenomenon is commonly known as the dynamic failure of the shock body.

#### Stability prediction of key load-bearing circle of surrounding rock

With the increase in mining depth, the stress of the surrounding rock increases continuously until it becomes unstable and is destroyed, causing rock burst. Therefore, the catastrophe model function $P_{0} (\Delta \sigma_{z\max } )$ of the maximum stress increment $\Delta \sigma_{z\max }$ and the rock stress $P_{0}$ is established. According to the standard potential function of the cusp catastrophe model, the following polynomials can be constructed

24 $$ \Delta \sigma_{z\max } = a_{0} + a_{1} P_{0} + a_{2} P_{0}^{2} + a_{3} P_{0}^{3} + a_{4} P_{0}^{4} , $$

where $a_{1}$, $a_{2}$, $a_{3}$, and $a_{4}$ are the undetermined coefficients.

When Eq. (24) is applied to the Tschirnhaus transformation principle, the specific process is.

When $P_{0} = y - Q$, $Q = \frac{{a_{3} }}{{4a_{4} }}$, Eq. (24) changes to

25 $$ \Delta \overline{\sigma }_{z\max } = b_{0} + b_{1} y + b_{2} y^{2} + b_{4} y^{4} , $$

where $$\left\{ {\begin{array}{*{20}c} {b_{0} } \\ {b_{1} } \\ {b_{2} } \\ {b_{4} } \\ \end{array} } \right\} = \left\{ {\begin{array}{*{20}c} {Q^{4} } & { - Q^{3} } & {Q^{2} } & { - Q} & 1 \\ { - 4Q^{3} } & {3Q^{2} } & { - 2Q} & 1 & 0 \\ {6Q^{2} } & { - 3Q} & 1 & 0 & 0 \\ 1 & 0 & 0 & 0 & 0 \\ \end{array} } \right\}\left\{ {\begin{array}{*{20}c} {a_{4} } \\ {a_{3} } \\ {a_{2} } \\ {a_{1} } \\ {a_{0} } \\ \end{array} } \right\}$$.

When $V = \Delta \sigma_{z\max } /4b_{4} ,u = b_{2} /4b_{4} ,v = b_{1} /4b_{4} ,c = b_{0} /4b_{4}$, Eq. (25) changes to

26 $$ V = \frac{1}{4}y^{4} + \frac{1}{2}uy^{2} + vy + c, $$

where $u,v$ are the control variable factors (the comprehensive index is dimensionless under the influence of multiple factors), $y$ is the state factor, and $c$ is the shear term, which is omitted, as it is meaningless for mutation analysis.

The standard form of the cusp catastrophe model is represented by Eq. (25). According to the theory of this model, the catastrophe threshold is defined as $\Delta = 4u^{3} + 27v^{2}$. When $\Delta = 0$, $V$ is the critical state of the stable equilibrium and imbalance. When $\Delta > 0$, $V$ is the stable equilibrium. When $\Delta < 0$, $V$ is the unstable equilibrium. The stability of $V$ is consistent with the stability of $\Delta$, which implies that the criteria for evaluating the stability of the roadway surrounding rock are as follows:

1. When $\Delta > 0$, it signifies that the roadway surrounding rock is in a state of stable equilibrium.
2. When $\Delta < 0$, it signifies that the surrounding rock of the roadway is in an unstable equilibrium.
3. When $\Delta = 0$, it signifies that the roadway surrounding rock is in a critical state.

**Figure 6:**
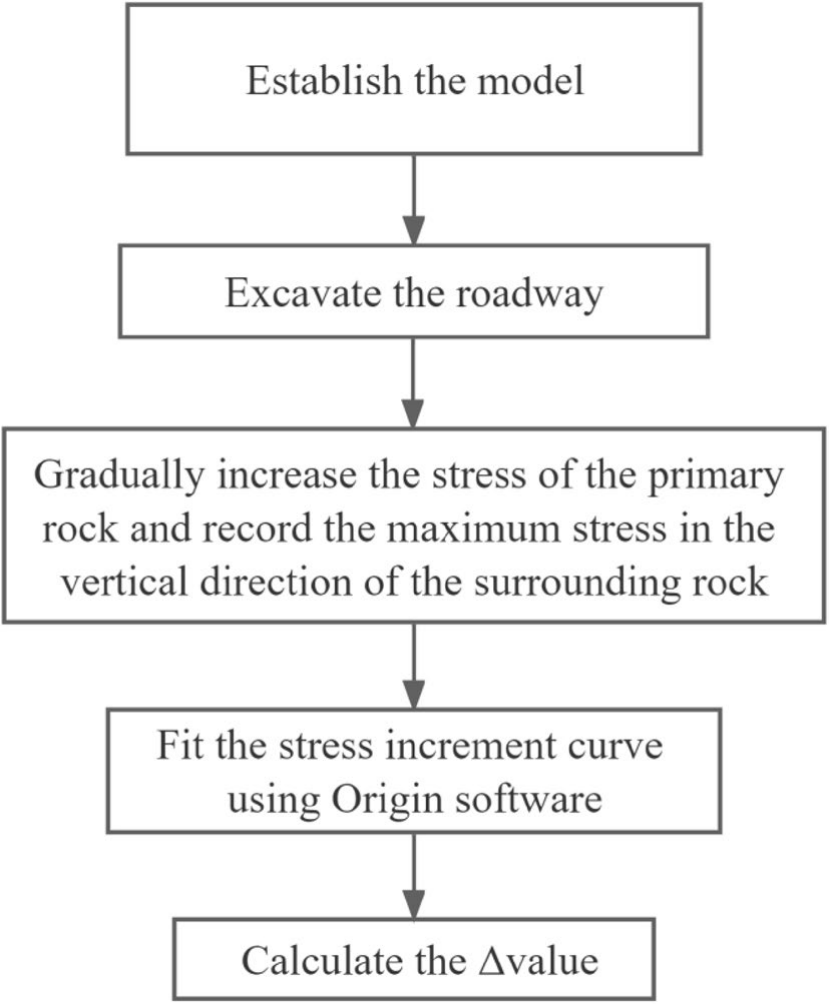
Analysis calculation steps.

The specific analysis steps are shown in Fig. 6.

### Example verification

#### Example analysis

**Figure 7:**
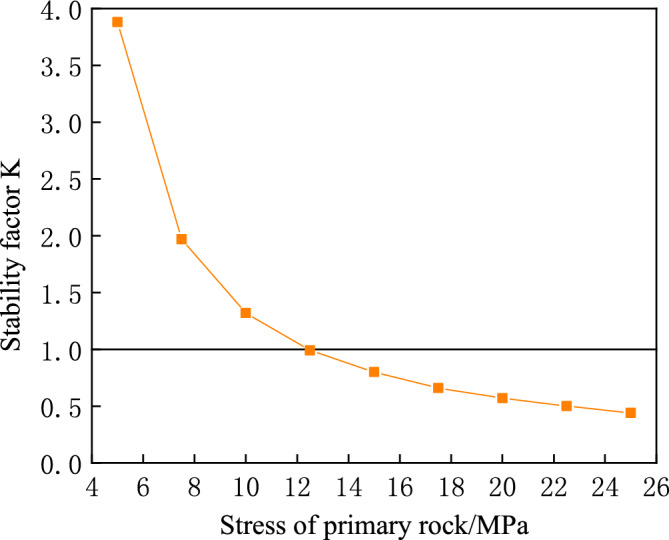
Stability coefficient under different primary rock stresses.

The physical and mechanical indices of the roadway surrounding rock are presented in Table 1^18^. Since rock bursts usually occur in areas where the surrounding rock is harder, the parameters for the Class III category of surrounding rock are used as the basis for calculation, with an excavation radius of 3 m for the tunnel^19^. The surrounding rock parameters can be inserted into Eq. (23) for analysis. According to Fig. 7, there is a continuous decrease in the surrounding rock stability coefficient as the stress of the primary rock increases. When the stress of the primary rock is greater than 12.5 MPa, the stability coefficient becomes less than 1, which demonstrates that the key load-bearing circle system of the surrounding rock is in unstable equilibrium and the roadway experiences rock burst.

**Table 1:** Physical and mechanical indexes of surrounding rock.

Elastic modulus/GPa	Cohesion/MPa	Friction angle/°	Poisson's ratio
13	1.1	45	0.275

#### Model building

**Figure 8:**
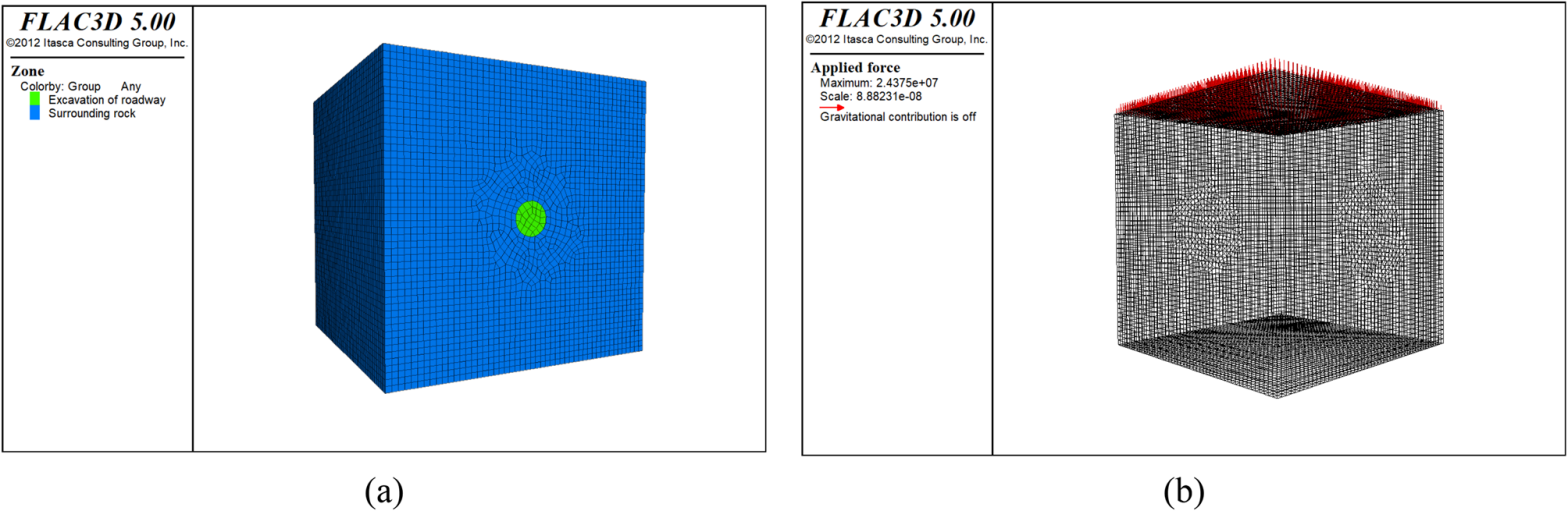
Numerical calculation model: (**a**) Model grid division and grouping, (**b**) Scope of stress application.

The engineering model depicted in Fig. 8 is specifically developed to examine the deformation boundary of the foundational model across different primary rock stress conditions. The model has dimensions of 50 × 50 × 50 m and employs a Cartesian 3D coordinate system to establish the boundary conditions. Normal constraint boundaries are applied to the bottom, left, right, front, and rear portions of the model. The construction of the model incorporates the Mohr–Coulomb material model.

#### Interpretation of result

**Figure 9:**
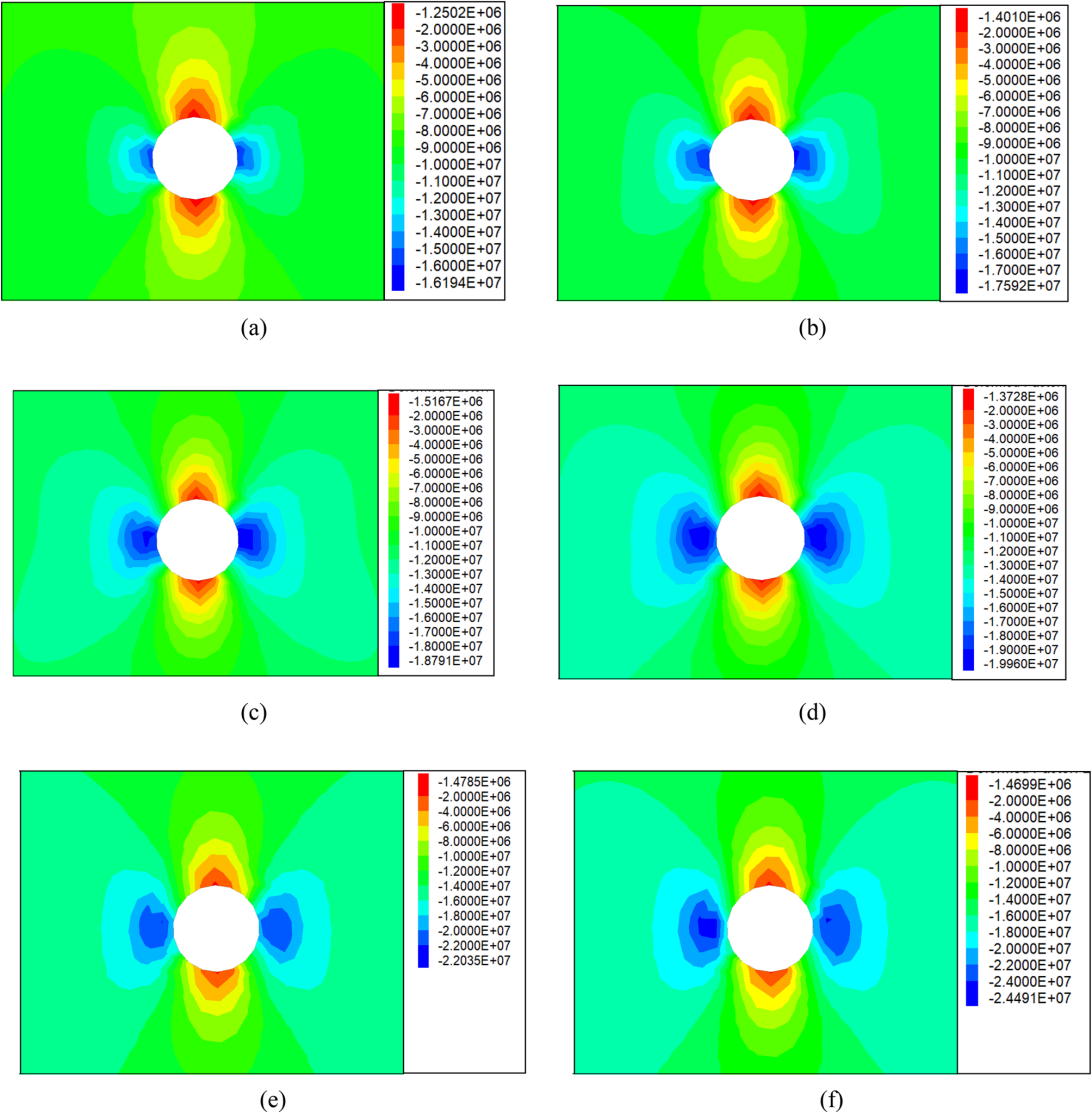
Vertical stress cloud map of surrounding rock under different primary rock stresses: (**a**) Original rock stress: 8.75 MPa, (**b**) Original rock stress: 10 MPa, (**c**) Original rock stress: 11.25 MPa, (**d**) Original rock stress: 12.5 MPa, (**e**) Original rock stress: 13.75 MPa, (**f**) Original rock stress: 15 MPa.

**Figure 10:**
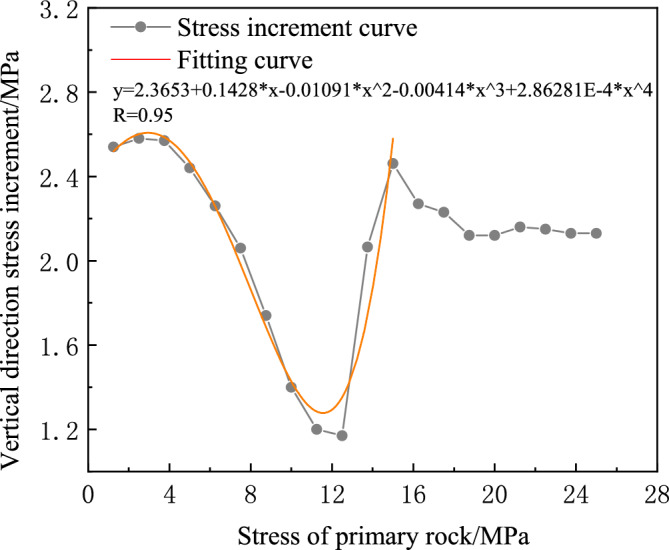
Stress increment curve fitting.

The numerical simulation reveals the maximum stress values of the surrounding rock in the vertical direction under different primary rock stresses, as illustrated in Fig. 9. Due to space limitations, only the results for the range of 8.75–15 MPa are provided. These simulation findings are summarized in Table 2 for easy reference. Moreover, by following the process steps outlined in Fig. 6, the maximum stress increments in the vertical direction of the surrounding rock are fitted under varying primary rock stress conditions, as depicted in Fig. 10.

**Table 2:** Maximum stress and stress increment in the vertical direction for different primary rock stresses.

Stress of primary rock /${\text{MPa}}$	Maximum stress in the vertical direction /${\text{MPa}}$	Stress increment /${\text{MPa}}$
1.25	2.54	2.54
2.50	5.12	2.58
3.75	7.69	2.57
5.00	10.13	2.44
6.25	12.39	2.26
7.50	14.45	2.06
8.75	16.19	1.74
10.00	17.59	1.40
11.25	18.79	1.20
12.50	19.96	1.17
13.75	22.03	2.07
15.00	24.49	2.46
16.25	26.76	2.27
17.50	28.99	2.23
18.75	31.11	2.12
20.00	33.23	2.12
21.25	35.39	2.16
22.50	37.54	2.15
23.75	39.67	2.13
25.00	41.80	2.13

The origin software was used to fit the surrounding rock stress increment. The values $a_{0}$, $a_{1}$, $a_{2}$, $a_{3}$, $a_{4}$, $u$, $v$, and $\Delta$ are calculated using Eq. (25). Subsequently, an assessment is made to determine if there is a possibility of catastrophic change in the surrounding rock based on the $\Delta$-value. To ensure a clear presentation of the calculation results, they are then displayed in Table 3. Table 3 shows that the catastrophe characteristic value $\Delta$ varies with the stress of the primary rock at 15 MPa, being either positive or negative. This indicates that the key load-bearing circle system is in an unstable equilibrium state. Consequently, this instability leads to rock burst. This demonstrates that the instability results after applying the instability mechanical criterion of the load-bearing circle system are consistent with the numerical simulation results. Hence, the correctness of the mechanical instability criterion is verified.

**Table 3:** Parameter calculation results.

Stress of primary rock /${\text{MPa}}$	a0	a1	a2	a3	a4	u	v	Δ	Catastrophe discrimination
1.25	1.000	1.000	1.000	1.000	1.000000	0.156	0.156	$> 0$	Stabilization
2.50	1.000	1.000	1.000	1.000	1.000000	0.156	0.156	$> 0$	Stabilization
3.75	1.000	1.000	1.000	1.000	1.000000	0.156	0.156	$> 0$	Stabilization
5.00	1.000	1.000	1.000	1.000	1.000000	0.156	0.156	$> 0$	Stabilization
6.25	1.000	1.000	1.000	1.000	1.000000	0.156	0.156	$> 0$	Stabilization
7.50	2.510	−0.014	0.045	−0.013	0.000853	−9.331	−18.174	$> 0$	Stabilization
8.75	2.339	0.230	−0.064	0.006	−0.000264	16.622	119.524	$> 0$	Stabilization
10.00	2.384	0.169	−0.039	0.002	−0.000066	70.417	757.109	$> 0$	Stabilization
11.25	2.507	0.017	0.017	−0.006	0.000304	−22.440	−87.427	$> 0$	Stabilization
12.50	2.544	−0.026	0.032	−0.008	0.000384	−18.782	−74.623	$> 0$	Stabilization
13.75	2.833	−0.344	0.132	−0.020	0.000839	−11.991	−42.417	$> 0$	Stabilization
15.00	2.365	0.143	−0.011	−0.004	0.000286	−29.133	−38.695	$< 0$	Catastrophic Change

### Engineering case

The computational approach embodied in Eq. (22) was utilized to ascertain the theoretical critical mining depth pertaining to rock burst in several Chinese mines. A comparison of these theoretically-derived values with the values gathered from field observations is depicted in Table 4^3,20–26^. Owing to fluctuating degrees of geological structural stress within the coal seam environment, the theoretical values for the critical mining depth associated with rock burst incidents tend to exceed the values derived from actual engineering statistics. Notably, the discrepancy between the theoretical and measured statistical values was found to be less than 35%, while the difference between the proposed theory's critical depth calculation and the value determined by Pan Yishan's theory^3^ was less than 32%. Due to the complexity of the geological conditions encountered in coal mine engineering, a certain margin of error exists between theoretical calculations and statistical values. For rock burst predictions, the generally acceptable error range is usually within 20%^27–31^; however, in more complex situations, this range can extend to within 50%^32,33^. Such allowances further affirm the engineering application value of the theory.

**Table 4:** Comparison of measured and theoretical critical depths for rock burst occurrence in some coal mines.

	Name of coal mine	Elastic modulus $E({\text{GPa)}}$	Poisson's ratio $\mu$	Uniaxial compressive strength $\sigma_{{\text{c}}} ({\text{MPa)}}$	Critical mining depth				
					Theoretical values	Pan Yishan’s theoretical values	Measured statistical values	The discrepancy with the statistical values %	The discrepancy in the theory with Pan Yishan %
Qi	Longfeng Mine	3	0.3	9	376	351	350	6.91	6.65
	Tianchi Mine	2.4	0.28	12	424	418	395	6.84	1.42
	Mentougou Mine	8.2	0.29	14	302	397	200	33.77	31.46
	Taozhuang Mine	5.6	0.35	18	524	540	480	8.40	3.05
	Tangshan Mine	7.8	0.29	10	518	426	500	3.47	17.76
	Dongtan Mine	3.97	0.32	14.5	548	483	586	6.93	11.86
He	Changcun Mine	7	0.38	12.86	699	896	669	4.29	28.18
Cao	Zhangshuanglou Mine	5.04	0.38	19.66	697	910	850	21.95	30.56
Zhang	Dongbaowei Mine	2.23	0.35	11.47	762	919	686	9.97	20.60
Li	Hongyangsan Mine	4.1	0.14	7.51	817	990	1082	32.44	21.18
Wang	Zhaoxian Mine	5.94	0.27	13.47	925	880	792	14.38	4.86
Feng	Hongqinghe Mine	6.0	0.3	29.56	1209	1129	746	33.92	14.53
Zhao	Jixian Mine	6.08	0.3	7.33	823	871	689	16.28	5.83
Du	Yizhouyao Mine	2.29	0.243	26	742	737	550	25.87	0.67

Simultaneously, Eq. (21) is applicable for determining the critical load associated with rock burst events. The theoretical calculations of critical loads, based on the mechanical properties of coal seams from some rock burst-prone mines in China^2,20,34–38^, are illustrated in Table 5. From the table, it is evident that the critical load $P_{{{\text{cr}}}}$ increases with higher uniaxial compressive strength $\sigma_{{\text{c}}}$ of coal and rock. Moreover, the ratio of critical load to uniaxial compressive strength was observed to fall within the range of 0.38 to 1.93. This range is congruent with the empirical criteria applied to the engineering classification of rock masses, where a standard $P_{{{\text{cr}}}} > 0.{25}\sigma_{{\text{c}}}$ is considered^39^.

**Table 5:** Theoretical critical load values of rock burst occurrence in some coal mines.

	Name of coal mine	Elastic modulus $E({\text{GPa)}}$	Poisson's ratio $\mu$	Uniaxial compressive strength $\sigma_{{\text{c}}} ({\text{MPa)}}$	Critical load values $P_{{{\text{cr}}}} ({\text{MPa)}}$	Ratio of critical load to compressive strength $P_{{{\text{cr}}}} /\sigma_{{\text{c}}}$
Xuan	MenkeqingMine	0.35	0.32	28.01	10.51	0.38
Li	Huatai Mine	0.29	0.34	1.41	1.15	0.82
	Shennanao Mine	0.8	0.38	5.93	3.63	0.61
	Xin'an Mine	1.66	0.22	13.87	7.64	0.55
	Yuejin Mine	2.46	0.36	15.21	10.09	0.66
	Bayan Gaole Mine	2.22	0.28	28.57	13.69	0.48
Zhao	Jixian Mine	6.08	0.3	7.31	13.64	1.87
Du	Yizhouyao Mine	2.29	0.243	26	12.86	0.49
Gao	Xinjulong Mine	17.04	0.37	17.41	33.66	1.93
Dong	Wudong Mine	1.71	0.32	13.82	7.76	0.56
Qi	Huating Mine	5.32	0.37	12.4	15.28	1.23
	Huafeng Mine	8.33	0.35	22.6	24.68	1.09
	Sanjianhe Mine	8.9	0.32	23.9	25.83	1.08

## Conclusion

1. Utilizing catastrophe theory, a mechanical analysis model was established, incorporating geometric structural parameters, environmental loads, and physical and mechanical parameters of coal and rock mass. This model permits identification of the instability in the critical load-bearing circle system of roadway surrounding rock, while concurrently elucidating the theoretical formulas for rock burst's critical loads and depths. As a result, this model enables the quantitative calculation of the critical conditions requisite for rock burst initiation.
2. Utilizing the stress increment mutation criterion, a predictive model for roadway rock burst in the surrounding rock has been formulated, providing an instability discrimination formula. A comparative verification analysis was conducted based on case studies, confirming the validity of the instability of the key load-bearing circle system in the roadway surrounding rock.
3. Upon examining various real-world engineering scenarios, it was determined that the discrepancy between the calculated critical depth of this study and the statistically observed value is less than 35%. Moreover, the variation between the critical depth calculated in this study and the value obtained from Pan Yishan's theory is also less than 32%. Notably, the critical load ratio derived in this paper, relative to the uniaxial compressive strength, falls within the range of 0.38–1.93. This result is consistent with empirical experience concerning rock burst occurrence, as delineated in the standard for engineering classification of rock masses. Collectively, these outcomes further reinforce the pragmatic validity of the proposed theory.

## Data Availability

Data sets generated during the current study are available from the corresponding author on reasonable request.
